# Supplementary material for: A look-ahead Monte Carlo simulation method for improving parental selection in trait introgression
Source: Sci Rep. 2021 Feb 16;11:3918. doi: 10.1038/s41598-021-83634-x (PMC7887201; doi:10.1038/s41598-021-83634-x)
Supplement: Supplementary file 1 — Supplementary Information [file 41598_2021_83634_MOESM1_ESM.pdf]

Supplementary Information: A Look-ahead Monte Carlo Simulation Method for Improving Parental Selection in Trait Introgression

Saba Moeinizade[1], Ye Han[2], Hieu Pham[2], Guiping Hu[1,*], and Lizhi Wang[1]

[1]Industrial and Manufacturing Systems Engineering, Iowa State University, Ames, IA 50011, USA

[2]Syngenta, Slater, IA 50244, USA

[*]Corresponding Author: gphu@iastate.edu

1 Appendix

Recombination frequencies for 3 data sets that were used in this study.

	Ch1	Ch2	Ch3	Ch4	Ch5	Ch6	Ch7	Ch8	Ch9	Ch10
Marker1	0.0662	0.1441	0.0865	0.1588	0.0790	0.0915	0.0955	0.0662	0.1266	0.0722
Marker2	0.1812	0.1122	0.0412	0.0705	0.0739	0.0955	0.0782	0.0636	0.0748	0.1176
Marker3	0.0458	0.0574	0.1035	0.1067	0.0592	0.0627	0.1003	0.0610	0.0765	0.0915
Marker4	0.0357	0.0394	0.0979	0.1615	0.0282	0.0253	0.1152	0.0874	0.0906	0.0857
Marker5	0.0512	0.0671	0.1533	0.1051	0.0679	0.0739	0.0955	0.0857	0.1325	0.0731
Marker6	0.0627	0.0310	0.1682	0.0849	0.0060	0.0857	0.0987	0.0592	0.0773	0.0234
Marker7	0.0167	0.0347	0.0697	0.0748	0.0610	0.0857	0.0263	0.0636	0.0636	0.0739
Marker8	**0.0089**	0.0282	0.0697	0.0748	0.0557	0.0503	0.0688	0.1398	0.1145	0.1003
Marker9	0.0050	0.0158	0.1413	0.0636	0.0215	0.0565	0.1176	0.0840	0.1347	0.0592
Marker10	0.0089	**0.0089**	0.0565	0.1688	0.0070	0.0412	0.0653	0.0557	0.0748	0.0412
Marker11	0.0662	0.0253	0.0857	0.0815	0.0530	0.1377	0.1090	0.0282	0.0653	0.0494
Marker12	0.0476	0.0010	0.0583	0.0756	**0.0020**	0.0412	0.0679	0.0512	0.0476	
Marker13	0.0244	0.1333	0.0939	0.0739	0.0196	0.1484	0.0627	0.1281	—	—
Marker14	0.1581	0.0040	0.0244	0.0244	0.0119	0.0301	—	0.1377	—	—
Marker15	0.1051	0.0722	0.0618	0.0512	0.0010	0.0645	—	0.0440	—	—
Marker16	0.0494	0.0430	0.0636	—	0.0375	—	—	0.0394	—	—
Marker17	0.1391	0.0282	0.0832	—	0.0731	—	—	—	—	—
Marker18	0.1191	0.0476	0.0748	—	0.0301	—	—	—	—	—
Marker19	0.0995	0.0782	0.0366	—	0.0583	—	—	—	—	—
Marker20	0.0662	0.0476	—	—	0.0931	—	—	—	—	—
Marker21	0.1470	0.0947	—	—	0.0421	—	—	—	—	—
Marker22	0.0539	0.0618	—	—	0.0319	—	—	—	—	—
Marker23	0.0931	0.1075	—	—	0.0653	—	—	—	—	—
Marker24	0.0765	0.0253	—	—	0.2045	—	—	—	—	—
Marker25	0.1011	0.0583	—	—	0.0874	—	—	—	—	—
Marker26	0.0748	0.0722	—	—	0.0782	—	—	—	—	—
Marker27	0.0714	—	—	—	0.0739	—	—	—	—	—
Marker28	0.0782	—	—	—	—	—	—	—	—	—
Marker29	0.0565	—	—	—	—	—	—	—	—	—
Marker30	0.0592	—	—	—	—	—	—	—	—	—
Marker31	0.0548	—	—	—	—	—	—	—	—	—

Table 1: Recombination frequencies for case 1. Each row refers to a marker and each column refers to a chromosome. The recombination frequencies for the three markers that should be integrated from the donor to the recipient are distinguished with an underline.

	Ch1	Ch2	Ch3	Ch4	Ch5	Ch6	Ch7	Ch8	Ch9	Ch10
Marker1	0.0799	0.0485	0.0674	0.0733	0.0028	0.0910	0.0757	0.0855	0.0770	0.0524
Marker2	0.0953	0.0626	0.0618	0.0614	0.0801	0.1472	0.1118	0.0966	0.0724	0.0671
Marker3	0.1057	0.1189	0.1467	0.0990	0.1353	0.0246	0.0587	0.0498	0.0793	0.0959
Marker4	0.0484	0.0868	0.1368	0.0406	0.0279	0.0814	0.0870	0.1510	0.0721	0.0914
Marker5	0.0911	0.0659	0.1306	0.1580	0.0236	0.1506	0.0928	0.1049	0.0894	0.0567
Marker6	0.0040	0.0693	0.0752	0.1174	0.0130	0.0737	0.0861	0.0888	0.0820	0.1009
Marker7	0.0792	0.0392	0.1079	0.0551	0.0113	0.1709	0.0640	0.0915	0.1219	0.1118
Marker8	0.0815	0.1110	0.1199	0.1113	**0.0150**	0.0663	0.1220	0.0966	0.0598	0.0510
Marker9	0.1409	0.1297	0.0646	0.0625	0.0129	0.1496	0.1310	0.0016	0.1178	0.0025
Marker10	0.1164	0.0997	0.1395	0.0790	0.0484	0.0285	0.0739	**0.0258**	0.0979	0.1216
Marker11	0.0670	0.0086	0.1265	0.0975	0.0115	0.0650	0.1451	0.0033	0.0545	0.0966
Marker12	0.1578	0.1076	0.1031	0.0975	0.0481	—	—	0.0146	0.0595	—
Marker13	0.1389	0.0115	0.0823	0.1265	0.0775	—	—	0.0645	—	—
Marker14	0.0542	0.1247	0.1043	0.1357	0.0922	—	—	0.0653	—	—
Marker15	0.0356	0.0729	—	—	0.0899	—	—	0.0541	—	—
Marker16	0.1474	0.0843	—	—	0.1107	—	—	0.1075	—	—
Marker17	0.0213	0.0833	—	—	0.0716	—	—	0.0450	—	—
Marker18	0.0938	0.0365	—	—	0.0923	—	—	—	—	—
Marker19	0.0527	—	—	—	0.0657	—	—	—	—	—
Marker20	0.0311	—	—	—	0.1572	—	—	—	—	—
Marker21	0.0490	—	—	—	0.0714	—	—	—	—	—
Marker22	0.0269	—	—	—	0.0853	—	—	—	—	—
Marker23	**0.0279**	—	—	—	0.0670	—	—	—	—	—
Marker24	0.0080	—	—	—	0.0653	—	—	—	—	—
Marker25	0.0257	—	—	—	0.1101	—	—	—	—	—
Marker26	0.0890	—	—	—	—	—	—	—	—	—
Marker27	0.0490	—	—	—	—	—	—	—	—	—
Marker28	0.1303	—	—	—	—	—	—	—	—	—
Marker29	0.0515	—	—	—	—	—	—	—	—	—
Marker30	0.0636	—	—	—	—	—	—	—	—	—

Table 2: Recombination frequencies for case 2.

	Ch1	Ch2	Ch3	Ch4	Ch5	Ch6	Ch7	Ch8	Ch9	Ch10
Marker1	0.2098	0.0722	0.0653	0.2332	0.0421	0.0697	0.1635	0.0697	0.0338	0.0756
Marker2	0.0739	0.1708	0.0384	0.0756	0.1333	0.0592	0.0756	0.0722	0.0557	0.0748
Marker3	0.0476	0.1695	0.0790	0.0503	0.0890	0.0906	0.1035	0.1027	0.0653	0.0874
Marker4	0.0440	0.0128	0.0530	0.1615	0.0592	0.0583	0.1377	0.1888	0.1019	0.1355
Marker5	0.0030	0.0225	0.0512	0.1003	0.0119	0.0739	0.0882	0.0234	0.0539	0.0865
Marker6	**0.0060**	0.0282	0.0060	0.1214	0.0574	0.1648	0.1662	0.1760	0.0987	0.1122
Marker7	0.0050	0.0158	0.1512	0.0731	0.0030	0.1251	0.0679	0.0565	0.0244	0.1051
Marker8	0.0089	**0.0089**	0.1152	0.1129	0.0592	0.0583	0.1027	0.0467	0.0557	0.0756
Marker9	0.0347	0.0263	0.1075	0.0955	0.0375	0.0898	—	0.0177	0.0601	0.0310
Marker10	0.0329	0.0291	0.0756	0.1183	0.0449	0.0688	—	0.1533	0.0898	0.0476
Marker11	0.0874	0.0206	0.0688	0.1043	0.0070	0.0548	—	0.0548	0.1405	—
Marker12	0.0196	0.0963	0.0291	0.0679	0.0530	0.0799	—	0.1413	0.1137	—
Marker13	0.1601	0.1244	0.0923	0.0329	**0.0020**	0.0301	—	0.0020	—	—
Marker14	0.0857	0.0099	0.1051	—	0.0196	0.0601	—	0.0485	—	—
Marker15	0.0244	0.1145	0.1831	—	0.0128	0.0244	—	—	—	—
Marker16	0.0824	0.1628	0.1427	—	0.0384	—	—	—	—	—
Marker17	0.1498	0.1296	0.0832	—	0.0440	—	—	—	—	—
Marker18	0.0375	0.1289	0.0748	—	0.0310	—	—	—	—	—
Marker19	0.0815	—	—	—	0.0824	—	—	—	—	—
Marker20	0.0618	—	—	—	0.0263	—	—	—	—	—
Marker21	0.1574	—	—	—	0.0739	—	—	—	—	—
Marker22	0.0119	—	—	—	0.0705	—	—	—	—	—
Marker23	0.0530	—	—	—	0.2767	—	—	—	—	—
Marker24	0.1114	—	—	—	0.0512	—	—	—	—	—
Marker25	0.0773	—	—	—	0.1051	—	—	—	—	—
Marker26	0.1168	—	—	—	—	—	—	—	—	—
Marker27	0.1281	—	—	—	—	—	—	—	—	—
Marker28	0.1137	—	—	—	—	—	—	—	—	—
Marker29	0.0539	—	—	—	—	—	—	—	—	—

Table 3: Recombination frequencies for case 3.
